# HMGN2, a new anti-tumor effector molecule of CD8^+^ T cells

**DOI:** 10.1186/1476-4598-13-178

**Published:** 2014-07-25

**Authors:** Lin Su, Ankang Hu, Yang Luo, Wenjie Zhou, Ping Zhang, Yun Feng

**Affiliations:** 1 State Key Laboratory of Oral Diseases, West China College of Stomatology, Sichuan University, Chengdu, Sichuan, China

**Keywords:** HMGN2, PMBC, CD8^+^ T cells, Antitumor activity, Anti-tumor effector molecule

## Abstract

**Background:**

Cytolytic T lymphocytes (CTL) and natural killer (NK) cells have been implicated as important cells in antitumor responses. Our previous research has shown that high mobility group nucleosomal-binding domain 2 (HMGN2) could be released by IL-2 and PHA stimulated peripheral blood mononuclear cells (PBMCs) and also induced tumor cells apoptosis at low doses. In this study, we isolated and cultured PBMCs and CD8^+^ T cells to analyze the expression and antitumor effects of HMGN2.

**Methods:**

PBMCs from healthy donors were isolated using Human Lymphocyte Separation tube. CD8^+^ T cells were separated from the PBMCs using MoFlo XDP high-speed flow cytometry sorter. Activation of PBMCs and CD8^+^ T cells were achieved by stimulating with Phytohemagglutinin (PHA) or tumor antigen. In addition, the methods of ELISA, intracellular staining, and fluorescence-labeling assays were used.

**Results:**

PHA induced PBMCs to release high levels of HMGN2, and CD8^+^ T cells was the major cell population in PBMCs that release HMGN2 after PHA activation. Tumor antigen-activated CD8^+^ T cells also released high levels of HMGN2. Supernatants of tumor antigen-activated CD8^+^ T cells were able to kill tumor cells in a dose-dependent manner. This antitumor effect could be significantly blocked by using an anti-HMGN2 antibody. Fluorescence-labeling assays showed that the supernatant proteins of activated CD8^+^ T cells could be transported into tumor cells, and the transport visibly decreased after HMGN2 was depleted by anti-HMGN2 antibody.

**Conclusions:**

These results suggest that HMGN2 is an anti-tumor effector molecule of CD8^+^ T cells.

## Background

Cytolytic T lymphocytes (CTLs) and Natural killer (NK) cells function as antitumor immune cells. CTLs and NK cells are rich in cytoplasmic granules. Upon degranulation, these cells release cytotoxic substances that act on target cells [1]. Granules in the cytoplasm of CTL contain perforin, granzymes, granulysin, additional effector molecules involved in the antitumor response and several uncharacterized components [2-5].

Previously, we isolated and purified an antimicrobial polypeptide from interleukin (IL)-2 and PHA stimulated human peripheral blood mononuclear cells (PBMCs) and identified the polypeptide as high mobility group nucleosomal binding domain 2 (HMGN2). When cultured PBMCs were stimulate with IL-2 and PHA, HMGN2 was expressed in the cytoplasm and then released into the supernatant [6]. HMGN2 is one of the most abundant non-histone nuclear proteins of vertebrates and invertebrates [7], and is a highly conserved nucleosomal protein thought to be involved in unfolding higher-order chromatin structures and facilitating transcriptional activation of mammalian genes [7]. However, until now, the biological role of this protein has not been fully defined, and some new research results have revealed that the protein has additional functions.

In this study, we isolated and cultured human PBMCs and separated CD8^+^ T cells from PBMCs. PBMCs and CD8^+^ T cells were then activated by PHA or tumor antigen (T-Ag), followed by analysis of HMGN2 protein expression and release. Our results showed that activated CD8^+^ T cells express high levels of HMGN2 protein, and HMGN2 protein in the culture supernatant of activated CD8^+^ T cells had proved had anti-tumor effect.

## Results

### PHA-activated T cells released high levels of HMGN2

**Figure 1:**
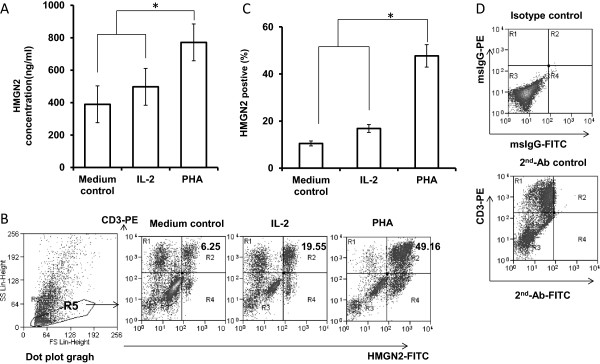
**PHA induced HMGN2 expression in PBMCs.** PBMCs were seeded at a density of 1 × 10^7^ per well in 6 well plates and stimulated with 20 μg/ml PHA or 100 IU/ml IL-2 for 72 hours. Normal medium was used as the control. **(A)** The supernatants were collected and HMGN2 concentrations were analyzed by ELISA. **(B)** The cells were removed and stained with anti-CD3-PE and anti-HMGN2-IgG-FITC, followed to run on a Beckman coulter FC500 Flow cytometry. Data were analyzed by using Submit 5.2 software after gate lymphocytes (R5) group on dot plot graph. Figures are representative of three independent experiments. **(C)** Error bars represent HMGN2 intracellular expression positive rate (%) in CD3^+^ T cells. Data are represented as means ± SD of three independent experiments. *Significantly higher compared to medium control (p < 0.05). **(D)** Representative plots of the isotype staining and 2^nd^-Ab-FITC staining control.

PHA is a lectin found in plants, especially legumes, and is a mitogen that triggers T-lymphocyte cell division and activation. Supernatants of PBMCs that have been stimulated by PHA contain a series of effector proteins. In our experiments, PBMCs were isolated from healthy donors and stimulated with 20 μg/ml PHA or 100 IU/ml IL-2 for 72 hours. Supernatants were collected and HMGN2 concentrations were analyzed by ELISA. The results showed that PBMCs release high levels of HMGN2 (771.33 ± 123.12 ng/ml) following 20 μg/ml PHA stimulation for 72 hours. Compared with the medium control (289.67 ± 98.5 ng/ml) and IL-2 stimulated (397.67 ± 134.15 ng/ml), HMGN2 concentration significantly increased (Figure 1A). In order to make sure that HMGN2 was released by activated T cells, we used surface and indirect intracellular staining to analyze HMGN2 expression in CD3^+^ T cells. Consistent with the ELISA results (Figure 1A), compared with the medium control (9.06 ± 3.8%) and IL-2 stimulated (18.85 ± 8.71%), the percentage of HMGN2^+^CD3^+^ cell population significantly increased after PHA stimulation (47.79 ± 11.87%) (Figure 1B and C).

### HMGN2 was highly expressed in activated CD8^+^ T cells

**Figure 2:**
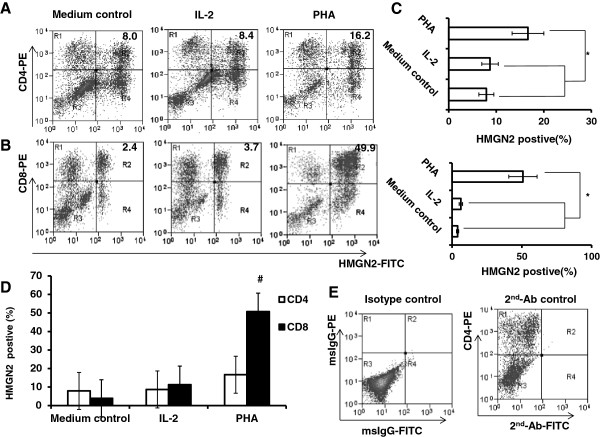
**PHA induced HMGN2 expression in different cell populations.** PBMCs were seeded at a density of 1 × 10^7^ per well in 6 well plates and stimulated with 20 μg/ml PHA or 100 IU/ml IL-2 for 72 hours. Normal medium was used as the control. Cells were collected and stained **(A)** Anti-CD4-PE surface stained and anti-HMGN2/IgG-FITC intracellular stained; **(B)** Anti-CD8-PE surface stained and anti-HMGN2/IgG-FITC intracellular stained. Figures are representative of three independent experiments. **(C, D)** Error bars represent HMGN2 intracellular expression positive rate (%) in CD4^+^ or CD8^+^ T cell populations. Data are represented as means ± SD of three independent experiments. *Significantly higher compared to IL-2 and medium control (p < 0.05), #Significantly higher compared to CD4^+^ T cell population (p < 0.05). **(E)** Representative plots of the isotype staining and 2^nd^-Ab-FITC staining control.

**Figure 3:**
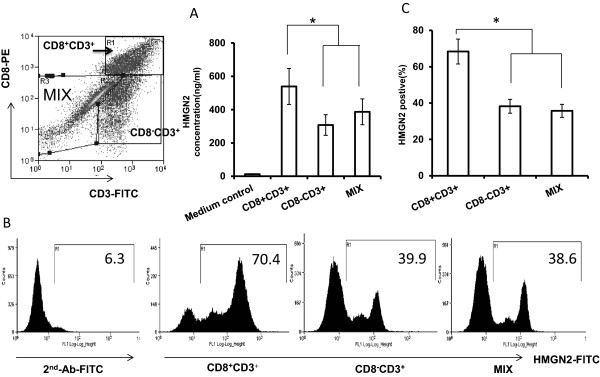
**PHA-induced HMGN2 was mainly expressed in CD8**^**+ **^**T cell population.** PBMCs were seeded at a density of 1 × 10^7^ per well in 6 well plates and stimulated with 20 μg/ml PHA for 72 hours. Cells were collected and stained with CD3-FITC and CD8-PE. CD3^+^CD8^+^T cells (Gate R1), CD3^+^CD8^-^T cells (Gate R2) and CD3^-^ mixed cells (Gate R3) were purified with MoFlo XDP sorter. 4 × 10^6^ CD3^+^CD8^+^T cells, CD3^+^CD8^-^T cells and CD3^-^ mixed cells were incubated in 24-well plates respectively for 5 days. **(A)** The supernatants were collected and HMGN2 concentrations were analyzed by ELISA. **(B)** The cells were removed and intracellularly stained for HMGN2. Figures are representative of three independent experiments. **(C)** Error bars represent HMGN2 intracellular expression positive rate (%) in CD8^+^ T cells populations. Data are represented as means ± SD of three independent experiments. *Significantly higher compared to CD8^-^CD3^+^ and MIX cells (p < 0.05).

To determine which T cell population expressed HMGN2, we stained the PHA pre-activated PBMCs with CD4-PE or CD8-PE surface staining. We then stained intracellular HMGN2 with rabbit anti-human HMGN2/goat anti-rabbit IgG-FITC. Results showed that HMGN2 expression significantly increased in both CD4^+^ and CD8^+^ T cell populations (Figure 2A, B, C). In addition, HMGN2 expression in CD8^+^ T cells (50.71 ± 10.34%) was significantly higher than that in CD4^+^ T cells (16.67 ± 5.61%) after PHA stimulation (Figure 2D). Finally, we used a MoFlo XDP high-speed flow cytometry sorter to purify PHA pre-activated CD3^+^CD8^+^ T cells, CD3^+^CD8^-^ T cells and CD3^-^ mix cells, followed by culturing in complete medium with 2000 IU/ml IL-2 for 5 days and analyzed the expression of HMGN2 by ELISA and intracellular staining. Results showed that HMGN2 was still expressed at high levels in CD8^+^ T cells (539.00 ± 118 ng/ml; 68.37 ± 15.21%). On the other hand, the expression of HMGN2 in the CD3^+^CD8^-^ T cells (mainly CD4^+^ T cells) (307.67 ± 97.34 ng/ml; 36.23 ± 11.52%) and the CD3^-^ mixed PBMCs population (386.67 ± 105.46 ng/ml; 35.73 ± 13.71%) was significantly lower in both supernatants and intercellular (Figure 3A, B and C). These results confirmed that activated CD8 + T cells expressed high levels of HMGN2.

### Tumor antigen activated CD8^+^ T cells expressed high levels of HMGN2

**Figure 4:**
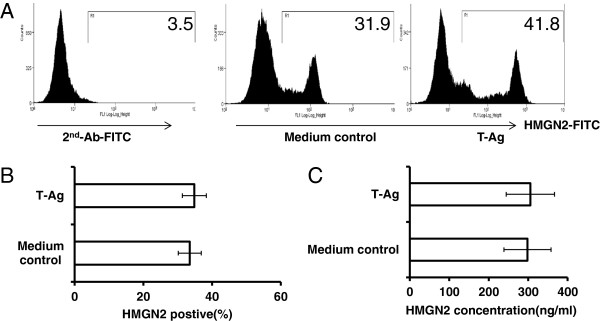
**HMGN2 expression in Tumor antigen stimulated PBMCs.** PBMCs were seeded at a density of 1 × 10^7^ per well in 6 well plates and stimulated with 150 μg/ml T-Ag for 7 days. Normal medium was used as the control. **(A)** The cells were removed and intracellularly stained for HMGN2. Figures are representative of three independent experiments. **(B)** Error bars represent HMGN2 intracellular expression positive rate (%) in PBMCs after stimulation with T-Ag. **(C)** The supernatants were collected and HMGN2 concentrations were analyzed by ELISA. Data are represented as means ± SD of three independent experiments.

**Figure 5:**
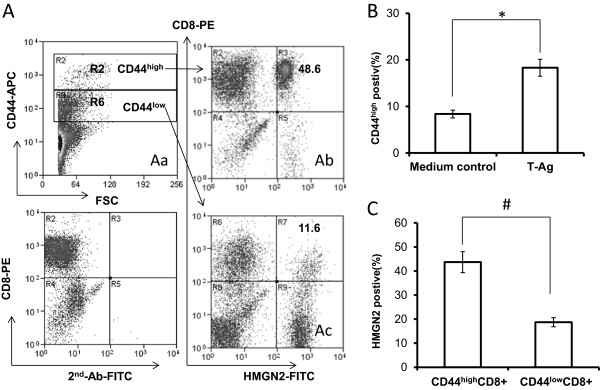
**T-Ag activated T cells and induced HMGN2 expression in activated T cell populations.** PBMCs were seeded at a density of 1 × 10^7^ per well in 6 well plates and stimulated with 150 μg/ml T-Ag for 7 days. Normal medium was used as the control. Cells were removed and (A) stained with CD44-APC/CD8-PE/intracellular HMGN2-2^nd^-Ab-FITC. **(Aa)** Gate 1 (CD44^high^) as the activated T cells, gate R6 (CD44^low^) as the naïve T cells. (Ab-Ac) Intracellularly expression of HMGN2 in CD44^high^ activated CD8^+^ T cells **(Ab)** and CD44^low^ naïve CD8^+^ T cells **(Ac)**. **(B)** Error bars represented the percentage of activation PBMCs after stimulated with T-Ag. **(C)** Error bars represented HMGN2 intracellular expression positive rate (%) in T-Ag-activated CD8^+^T cell populations. Data are represented as means ± SD of three independent experiments. *Significantly higher compared to medium control (p < 0.05). #Significantly higher compared to CD44^low^CD8^+^ naïve T cells (p < 0.05).

In order to ensure that HMGN2 was an effector protein of tumor antigen activated T cells, we used tumor full protein as tumor antigen (T-Ag) to stimulate PBMCs for 7 days. Supernatants were collected for assaying HMGN2 levels by ELISA and cells were collected for HMGN2 intracellular staining. Results showed that there was no significant change of HMGN2 expression after stimulation with T-Ag compared with the medium control (Figure 4). In order to ensure that T cells were activated by T-Ag, we used CD44^high^ as the activated marker of T cells. T-Ag stimulated PBMCs were stained with CD8-PE/CD44-APC surface and HMGN2 indirect intracellular staining. CD44^high^ was used as the activated T cell population (Figure 5Aa, gate R2) and CD44^low^ was used as the naive T cell population (Figure 5Aa, gate R6). T-Ag induced only 18.35 ± 6.20% PBMCs activated (Figure 5Aa, gate R2 and 5B). There was about 43.69 ± 12.51% of activated CD8^+^ T cells expressed HMGN2 (Figure 5Ab and C), where only 18.71 ± 6.34% naive CD8^+^ T cells expressed HMGN2 (Figure 5Ac and C).

**Figure 6:**
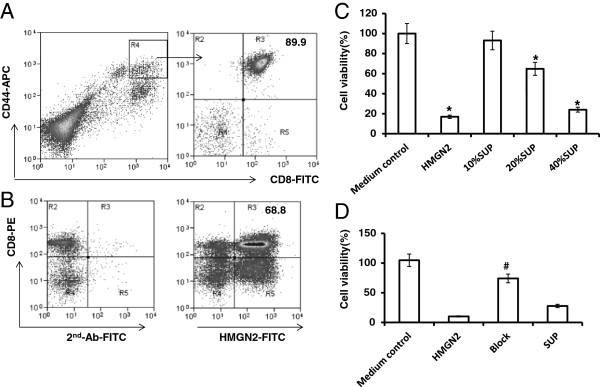
**T-Ag activated CD8**^**+**^**T cells released HMGN2 to kill tumor cells.** PBMCs were seeded at a density of 1 × 10^7^ per well in 6 well plates and stimulated with 150 μg/ml T-Ag for 7 days. **(A)** Cells were removed and stained with CD44-APC/CD8-FITC. CD44^high^/CD8^+^T activated T cells (Gate R4) were purified with MoFlo XDP sorter. The purified CD44^high^/CD8^+^T cells were cultured in complete medium with 2000 IU/ml IL-2 for 5 days. **(B)** The cells were removed and stained with Anti-CD8-PE surface stained and anti-HMGN2/IgG-FITC intracellular stained. **(C)** The antitumor effects of the supernatants at different concentration. **(D)** The antitumor effects of the 20% (v/v) supernatants after blocking HMHN2 using anti-HMGN2 antibody. Figures are representative of three independent experiments. Data are represented as means ± SD of three independent experiments. *Significantly decreased compared to medium control (p < 0.05). #Significantly decreased after with anti-HMGN2 antibody (p < 0.05).

We used a Beckman Coulter MoFlo XDP high-speed flow cytometry sorter to isolate T-Ag-activated the CD44^high^CD8^+^ T cell population (Figure 6A, Gate R4). The purified cells were cultured with 2000 IU/ml IL-2 for 5 days and then the cells were identified with CD44-APC and CD8-FITC staining (Figure 6A). Cells were collected and stained with CD8-PE surface and HMGN2 intracellular staining. A total of 69.35 ± 12.13% of CD8^+^ T cells still expressed HMGN2 (Figure 6B).

### HMGN2 as an anti-tumor effector molecule of CD8^+^ T cells

Tca8113 cells were seeded at a density of 1 × 10^3^ cells per well in 96-well plates. After overnight growth, the medium was replaced with maintenance medium containing the desired concentrations (v/v) of T-Ag activated CD8^+^ T cell supernatants. To one group, anti-HMGN2 antibody (10 μg/ml) was added, in order to block HMGN2. Human HMGN2 protein was used as the positive control, and the same volume of normal medium as the negative control. Cell viability was assessed after 72 hours using the CCK8 colorimetric assay. The supernatant was able to kill tumor cells in a dose-dependent manner; 20% supernatant could significantly kill tumor cells, as could the HMGN2 positive control (Figure 6C). The effect of killing tumor cells by the supernatant could be significantly inhibited by the anti-HMGN2 antibody (Figure 6D).

### HMGN2 protein could transmembrane transported into tumor cells

**Figure 7:**
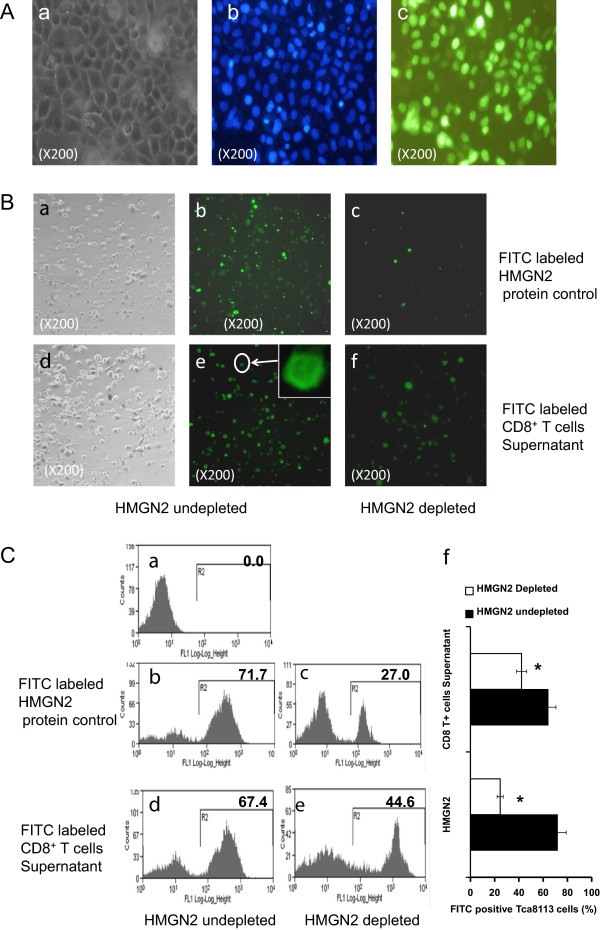
**HMGN2, released by T-Ag activated CD8**^**+ **^**T cells, transmembrane transported into tumor cells.** HMGN2 protein and the supernatant of T-Ag activated CD8^+^ T cells were pre-labeled with FITC. Tca8113 cells were seeded at a density of 3 × 10^4^ per well in 24-well plates. After overnight growth, the cells were cultured in medium with FITC pre-labeled samples. **(A)** HMGN2 transport into tumor cells analyzed with fluorescence microscope. The three figures are the same area. **(a)** Light micrographs of Tca8113 cells. **(b)** Fluorescent micrographs of Tca8113 cells of Hoechst 33258 nuclear staining. **(c)** Fluorescent micrographs of FITC labeled HMGN2 protein distribution in Tca8113 cells. **(B)** The Tca8113 cells were analyzed with fluorescent microscope. **(a, b, c)** FITC pre-labeled HMGN2 as the positive control. **(d, e, f)** FITC pre-labeled CD8^+^ T cells supernatant. **(a, d)** Cells under a light microscope. **(b, e)** Cells under a fluorescent microscope. **(c, f)** Cells under a fluorescent microscope after cultured in medium with HMGN2 depleted samples. **(C)** The Tca8113 cells were analyzed with Flow Cytometry. **(a)** Untreated Tca8113 control. **(b, d)** Tca8113 cultured in medium with FITC labeled samples. **(c, e)** Tca8113 cells cultured in medium with HMGN2 depleted samples. Figures are representative of three independent experiments. **(f)** Error bars represent FITC positive rate (%) of Tca8113 cells after cultured in medium with FITC labeled or HMGN2 depleted sample for 1 hour. Data are represented as means ± SD of three independent experiments. *Significantly decreased compared to HMGN2 undepleted (p < 0.05).

To confirm that the HMGN2 in the supernatant of T-Ag activated CD8^+^ T cells could be transmembrane transported into tumor cells, we used fluorescence FITC to label the proteins in the supernatant, before adding to the medium. Human FITC labeled HMGN2 protein was used as the positive control. Results showed that the protein in the supernatant could effectively be transported into the tumor cells, as could the human HMGN2 protein control (Figure 7A, Figure 7B b&e, Figure 7C b&d). After HMGN2-depleted by anti-HMGN2, the number of FITC-positive cells visibly decreased comparing with HMGN2 undepleted samples analyzed by both fluorescent microscope (Figure 7B b *vs* c, e *vs* f) and Flow Cytometry (Figure 7C b *vs* c, d *vs* e).

## Discussion

High mobility group (HMG) proteins have been described to be an abundant family of nonhistone proteins in cell nucleus of vertebrate and invertebrate organisms [7]. The HMG protein family is subdivided into three subfamilies: HMGB, HMGA and HMGN. Each subfamily appears to exert a single characteristic nuclear function [7]. However, peptides in the HMG protein family also exhibit adjunct roles. For example, HMGbox1 (HMGB1) is an abundant, highly conserved cellular protein, widely known as a nuclear DNA-binding protein [8,9]. A decade-long search has culminated in HMGB1 as a late toxic cytokine of endotoxemia. HMGB1, released by macrophages upon exposure to endotoxin, activates a number of other proinflammatory mediators and is lethal to otherwise healthy animals [8,9]. And, HMGB proteins 1, 2 and 3 had been found function as universal sentinels for nucleic-acid-mediated innate immune responses [10].

The HMGN family includes five chromatin architectural proteins that are present in higher vertebrates [11]. Of these proteins, HMGN1, 2, and 4 are expressed ubiquitously [12,13], whereas HMGN3 and 5 are expressed in specific tissues [14,15]. Initially, HMGNs were regarded as transcription co-regulators; their roles in DNA repair and cancer progression have, however, recently been established. Recent studies suggest that the archetype of HMGN1 has characteristics of a tumor suppressor gene [16]. In addition to HMGN1, the expression of HMGN5 (formerly NSBP1) was found to be elevated 4-fold in highly metastatic breast cancer cells compared with that in low metastatic cells [17]. In mice, overexpression of HMGN5 in the uterus was associated with the development of uterine adenocarcinoma [18,19]. These studies are consistent with the involvement of HMGN5 in cancer progression.

The HMGN2 gene is located at chromosome 1p36.1 and contains six exons [20], with an extremely high GC content and an “HpaII tiny fragment” island. These hallmarks are indicative of a housekeeping gene that may be crucial to the basal functioning of cells [7]. However, biological role of this protein has been poorly defined. HMGN2 is preferentially associated with chromatin subunits [7], and abnormal HMGN2 gene or protein expression is associated with development of neoplasms and autoimmune diseases [21,22]. Porkka et al. [23] examined phage-displayed cDNA libraries *in vivo* to search for phages capable of homing to the vascular endothelia of tumors. This revealed a remarkably potent homing peptide, F3, which corresponded to a 17–48 amino acid fragment in HMGN2. The 31-residue peptide was shown to selectively bind to tumor cells both *in vitro* and *in vivo*. And our previous showed that HMGN2 significantly inhibits the growth of Tca8113 cells, adenoid cystic carcinoma cell-2 line (ACC-2), human lung adenocarcinoma epithelial cell line A549 and bladder cancer cell line T24, which acted by promoting apoptosis *in vitro* and *in vivo*[24,25].

CTLs and NK cells are rich in cytoplasmic granules. Following degranulation, the cells release specific biologically active substances, which have a cytotoxic effect on target cells [1]. The granules in the cytoplasm of CTL contain perforin, granzymes, granulysin and other effector molecules involved in the anti-tumor effect, as well as certain unidentified components [2,3]. In our previous study [6], we found that HMGN2 is released by PBMCs in the presence of IL-2 and PHA. HMGN2 may represent an effector molecule for CTL or NK cells.

In the present study we isolated and cultured PBMCs and separated CD8^+^ T cells from PBMCs. PBMCs and CD8^+^ T cells were then activated by PHA or tumor antigen, followed by analysis of HMGN2 protein expression and release. Our results demonstrated an enhanced expression of HMGN2 protein in activated T cells, especially in activated CD8^+^ T cells. In addition, the supernatants of activated CD8^+^ T cells were able to kill tumor cells in a dose-dependent manner, and the tumor-killing effect of the supernatant could be significantly inhibited by anti-HMGN2 antibody. Fluorescence-labeling assays showed that HMGN2 in the supernatant of activated CD8^+^ T cells could be significantly transported into tumor cells. Our results suggest that HMGN2 is an anti-tumor effector molecule of CD8^+^ T cells.

## Conclusions

HMGN2 protein was enhanced express in activated T cells, especially in activated CD8^+^ T cells. In addition, HMGN2 protein in the culture supernatant of activated CD8^+^ T cells had antitumor effects. These results suggest that HMGN2 is an anti-tumor effector molecule of CD8^+^ T cells.

## Methods

### Preparation of T-Ag, activated T cells, and the cell culture supernatants

The human tongue squamous cell carcinoma cell line Tca8113 cells were obtained from the State Key Laboratory of Oral Disease (Sichuan University, Chengdu, China). Cells were cultured in RPMI 1640 medium supplemented with 10% FBS, 100 IU/ml penicillin, 100 μg/ml streptomycin, 3% L-glutamine, and 7.5% sodium bicarbonate (GIBCO Life Technologies). Cells were maintained as a monolayer in 25 cm plastic tissue culture flasks at 37°C in a humidified atmosphere containing 5% CO_2_. Tumor full protein (Tumor antigen, T-Ag) was prepared by lysing Tca8113 cells in PBS, the lysate was collected and stored at -80°C as T-Ag.

PBMCs from healthy human donors were isolated using Human Lymphocyte Separation tube (Beijing Dakewe Biotech Company Limited, China). PBMCs were plated at a density of 1 × 10^7^/well in six-well plates and cultured in RPMI 1640 medium containing 10% fetal bovine serum, 100 IU/ml penicillin, and 100 μg/ml streptomycin). The cells were stimulated with 150 μg/ml T-Ag for 7 days, or 20 μg/ml Phytohemagglutinin (PHA, Sigma, USA), 100 IU/ml IL-2 for 72 hours to activate T lymphocytes. Supernatants were collected and HMGN2 concentration was analyzed, along with its effect on tumor cell survival. Cells were removed to analyze HMGN2 expression by intracellular staining.

### Purifying T cell populations by flow cytometric sorting

PHA stimulated PBMCs were stained with CD8-PE/CD3-FITC at 4°C for 30 minutes. Cells were washed three times with sterilized PBS. The CD8^+^CD3^+^T cells, CD8^-^CD3^+^T cells, and CD3^-^ Mix cells populations were gated and isolated using MoFlo XDP high-speed flow cytometry sorter (Beckman). The purified T cell populations were cultured in complete medium with 2000 IU/ml IL-2 for 5 days. Cells were collected for analyzing HMGN2 expression.

T-Ag-stimulated PBMCs were stained with CD8-FITC/CD44-APC at 4°C for 30 minutes. CD44^high^CD8^+^ cells were gated as the activated CD8^+^ T cell population. CD44^high^CD8^+^ activated CD8^+^ T cells were isolated using a MoFlo XDP high-speed flow cytometry sorter (Beckman). The purified T cells were cultured in complete medium with 2000 IU/ml IL-2 for 5 days. Supernatants and cells were collected for analyzing anti-tumor effect and HMGN2 expression.

### ELISA analysis of HMGN2 concentration in the supernatant

ELISA plates were coated with 100 μl of supernatants, standards made from different concentrations of human HMGN2 protein, or PBS (negative control). The plates were left at 4°C overnight. After washing three times with wash buffer, 100 μl of rabbit anti-human HMGN2 antibody (Proteintech Group, USA) (1:500) were added and plates were incubated at 37°C for 1 hour. Then, 100 μl of HRP-conjugated anti-rabbit IgG secondary antibody (1:1000) were added after thorough washing of the first antibody, and plates were incubated at 37°C for 1 hour. Add 100 μl TMB substrate solution to each well. After 20 min incubation, reactions were stopped with 2 N H_2_SO_4_ and measured at 490 nm in an ELISA plate reader (VARIOSKAN FLASH, Thermo Fisher Scientific, Vantaa, Finland).

### Intracellular staining analysis of HMGN2 expression by flow cytometry

PHA or T-Ag stimulated PBMCs or purified T cell populations were collected and stained with fluorescence-labeled CD4, CD8, or CD44 surface marker or msIgG-PE/FITC isotypes (Biolegend, USA) control. After washing three times with wash buffer, cells were analyzed for HMGN2 expression by using an intracellular staining kit (Invitrogen, USA). Briefly, a volume of 100 μl fixation buffer was added to fix cells, which were then left at 4°C overnight, followed by washing with permeabilization buffer three times in order to permeabilize cells. All of the samples were divided into two tubes, rabbit-anti-human HMGN2 antibody (1 μg/ml) was added into one tube and the same volume PBS was added into the other as the 2^nd^-Ab-FITC control. The samples were incubated at 4°C for 1 hour. The cells were washed three times with permeabilization buffer and then incubated with goat anti-rabbit-IgG-FITC secondary antibody (2^nd^-Ab-FITC) at 4°C for 1 hour. Finally, the cells were washed with Flow Cytometry buffer and runed on a Beckman coulter FC500 Flow cytometry. Dates were analyzed by using Submit 5.2 software (Beckman Coulter, USA) after gate lymphocytes group on dot plot graph.

### Anti-tumor effects of HMGN2 in the supernatant of T-Ag-activated CD8^+^ T cells

Tca8113 cells were seeded at a density of 1 × 10^3^ cells per well in 96-well plates. After overnight growth, the medium was replaced with maintenance medium containing the desired concentrations (v/v) of supernatant of T-Ag-activated CD8^+^ T cells. Human HMGN2 protein was used as the positive control and medium as the negative control. Blocking of HMGN2 was achieved by adding 10 μg/ml anti-HMGN2 antibody to 20% (v/v) supernatants. Cell viability was assessed after 72 hours using the CCK8 colorimetric assay. Briefly, the cells were washed with 300 μl of PBS, followed by incubation with 100 μl of 5 mg/ml CCK8 in RPMI 1640 at 37°C for 1 hour, and quantified by measuring the optical absorbance (OA) at 450 nm in a plate reader (VARIOSKAN FLASH, Thermo Fisher Scientific, Vantaa, Finland).

### Fluorescence-labeled HMGN2 transmembrane transported assay

Human HMGN2 protein (2 μg/ml) and the supernatants of T-Ag activated CD8^+^ T cells were labeled with FITC. Briefly, FITC was added to 2 mg/ml protein solution and incubated for 5 hours at room temperature. The unconjugated FITC was removed using a 3 kDa filter. Half of these FITC-labeled samples were depleted of HMGN2 by using anti-human HMGN2 antibody adsorption in 96-well plates. Briefly, 10ug anti-human HMGN2 antibody was coated in 96-well plates at 4°C for overnight. The free antibody was removed by washing the wells with PBS three times. The FITC labeled samples were added into the wells and incubated at 37°C for 1 hour. The samples were collected and store at -80°C as the HMGN2 depleted samples.

Tca8113 cells were seeded at a density of 3 × 10^4^ per well in 24-well plates. After overnight growth, 2 μg FITC-labeled HMGN2, 20% (v/v) FITC-labeled CD8^+^ T cells supernatants and the same volume HMGN2-depleted samples were added to the mediums, respectively. Plates were incubated at 37°C in a humidified atmosphere containing 5% CO_2_ for 1 hour. Nuclear staining control of Tca8113 cells was measured using Hoechst 33258 (Promega Corporation, Madison, WI, USA). Staining was performed according to the manufacturer's instructions. Cells were analyzed under a fluorescence microscope and pictures were taken. Then, all the cells were removed with 0.25% trypsin and analyzed FITC positive cells on a Beckman coulter FC500 using Submit 5.2 software. Untreated Tca8113 cells were used as the negative control.

### Statistical analysis

All values were expressed as means ± SEM. Data were analyzed by one-way analysis of variance (ANOVA) followed by Bonferroni test. P < 0.05 was considered to indicate a statistically significant difference.

## Competing interests

The authors declare no competing interests.

## Authors’ contributions

LS and AH carried out the cell culture and separation, flow cytometry sorting and fluorescence-labeled assays. YL carried out the ELISA and intracellular staining studies. WZ collected and preserved samples. PZ and YF participated in the design of the study, performed the statistical analysis, and helped to draft the manuscript. All authors read and approved the final manuscript.
